# The mechanisms of a mammalian splicing enhancer

**DOI:** 10.1093/nar/gky056

**Published:** 2018-01-31

**Authors:** Andrew M Jobbins, Linus F Reichenbach, Christian M Lucas, Andrew J Hudson, Glenn A Burley, Ian C Eperon

**Affiliations:** 1 Leicester Institute of Structural & Chemical Biology and Department of Molecular & Cell Biology, University of Leicester, UK; 2 Department of Pure and Applied Chemistry, University of Strathclyde, UK; 3 Leicester Institute of Structural & Chemical Biology and Department of Chemistry, University of Leicester, UK

## Abstract

Exonic splicing enhancer (ESE) sequences are bound by serine & arginine-rich (SR) proteins, which in turn enhance the recruitment of splicing factors. It was inferred from measurements of splicing around twenty years ago that *Drosophila* doublesex ESEs are bound stably by SR proteins, and that the bound proteins interact directly but with low probability with their targets. However, it has not been possible with conventional methods to demonstrate whether mammalian ESEs behave likewise. Using single molecule multi-colour colocalization methods to study SRSF1-dependent ESEs, we have found that that the proportion of RNA molecules bound by SRSF1 increases with the number of ESE repeats, but only a single molecule of SRSF1 is bound. We conclude that initial interactions between SRSF1 and an ESE are weak and transient, and that these limit the activity of a mammalian ESE. We tested whether the activation step involves the propagation of proteins along the RNA or direct interactions with 3′ splice site components by inserting hexaethylene glycol or abasic RNA between the ESE and the target 3′ splice site. These insertions did not block activation, and we conclude that the activation step involves direct interactions. These results support a model in which regulatory proteins bind transiently and in dynamic competition, with the result that each ESE in an exon contributes independently to the probability that an activator protein is bound and in close proximity to a splice site.

## INTRODUCTION

The high point in studies on the mechanisms of action of exonic splicing enhancer sequences was reached in 1998. It had been shown previously that sequences in alternative exons were required for their inclusion (1–5), and in mammals these sequences had been mapped to relatively short, purine-rich motifs (6–8). These motifs were found to be bound by SR proteins (8–10). SR proteins have one or two RNA recognition motif (RRM)-type RNA-binding domains and a C-terminal RS domain, rich in arginine-serine dipeptides, that is subject to extensive phosphorylation (11–13). The binding of these proteins to ESEs was shown to stimulate splicing, splicing complex assembly and binding of U2 snRNPs and U2-associated proteins U2AF65 and U2AF35 (8,14–16). Moreover, SR proteins were shown also to interact directly with U2AF35 and this interaction was shown to mediate the effects of ESEs on the splicing of an upstream intron (17,18). These observations led to two models for the mechanisms of action of ESEs. ESEs that are within 100 nts or so of the 3’SS were proposed to work by binding of an SR protein to the ESE and propagation of the complex by cooperative interactions with further SR proteins until interactions with U2AF were possible; ESEs that are regulated, such as the repeated elements in *Drosophila dsx* exon 4 (*dsx*REs) that depend on the proteins Tra and Tra2, might form stable multi-protein complexes and interact directly over greater distances by 3D-diffusion (looping) (18).

**Figure 1.**
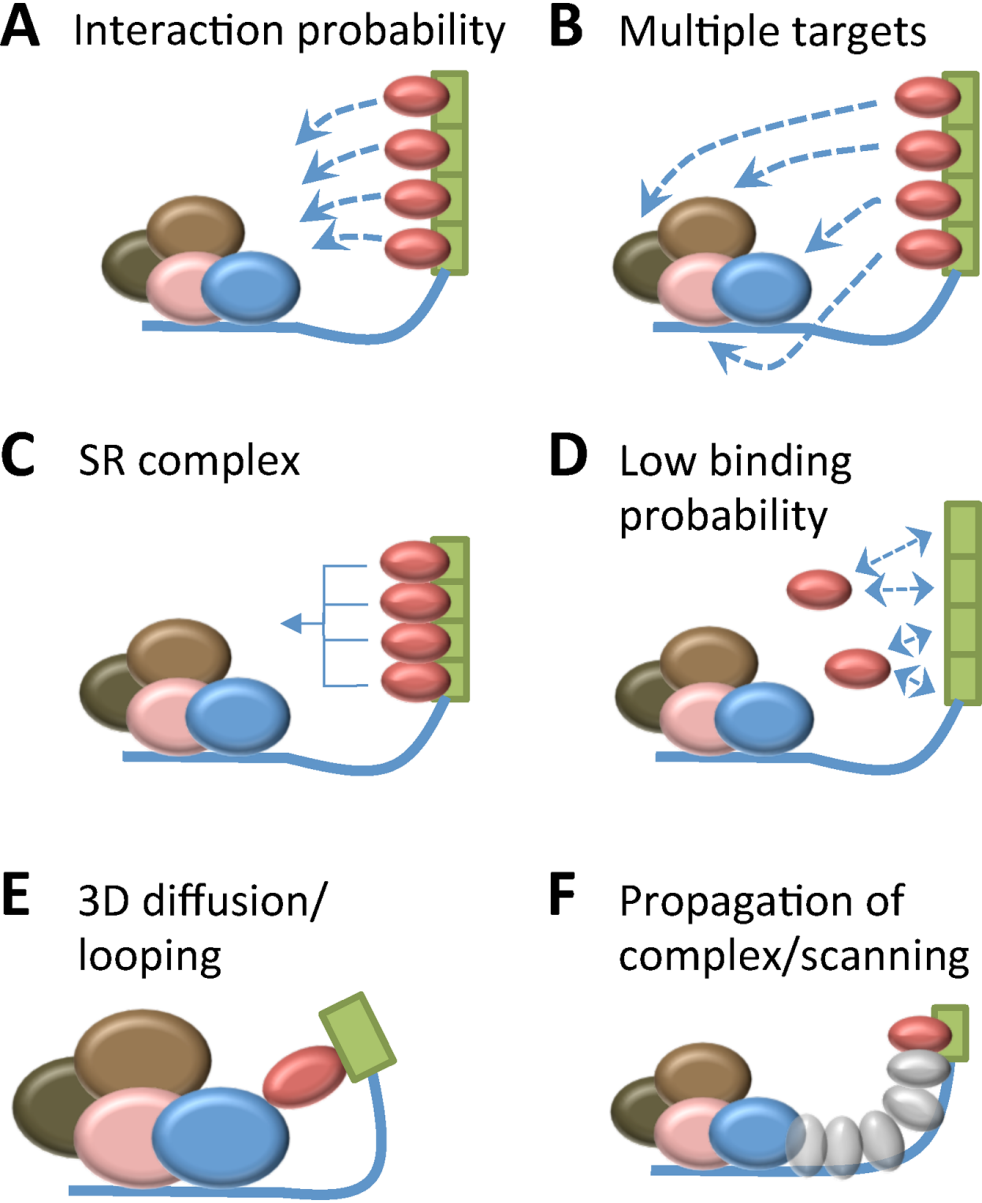
Elementary processes representing possible SR protein binding (A–D) and activation (E and F) steps by which ESEs stimulate 3′ splice site usage. (**A**) SR proteins bind stably, with multiple occupancy of tandemly-repeated ESEs, and the probability of subsequent interactions by mechanisms E or F is limiting. Solid blue line, pre-mRNA; red oval, SRSF1; other ovals, components at the 3’SS (U2AF, U2 snRNP, etc.); dashed lines, low probability interactions. (**B**) SR proteins bind stably, with multiple occupancy of tandemly-repeated ESEs, and are able to interact with multiple targets, each of which has an approximately equal effect on the rate. (**C**) SR protein binding is cooperative. (**D**) The probability of binding by an SR protein is very low and each site contributes independently. (**E**) Activation involves direct contact by three-dimensional diffusion between an ESE-bound SR protein and a 3’SS component; the intervening RNA is looped out. (**F**) Activation involves processes that maintain contact with the RNA between the ESE and the 3’SS, such as propagation of SR protein complexes or scanning along the RNA (for example, in conjunction with a helicase).

These models resolve the mechanisms of action of ESEs into two steps, either of which might be limiting or regulated. The first is binding to the ESE (Figure 1A–D); the second is the step in which the ESE-bound SR protein activates its target splice site (Figure 1E and F). The models were tested indirectly in two important papers in 1998 by following the rates of splicing dependent on the *dsx*REs (19,20). The proportion of *dsx* pre-mRNA that had spliced *in vitro* in a given time was shown to increase with the addition of Tra/Tra2, but the level of splicing asymptotically approached a maximum with increasing concentrations of protein. Assuming that the maximum level of splicing was a direct function of *dsx*RE occupancy and not, for example, the result of limiting solubility or aggregation at higher protein concentrations, it was inferred that the *dsx*REs had become saturated (20). Since the concentration of Tra/Tra2 required for half-maximal splicing did not depend on the number of *dsx*REs, it was concluded that each *dsx*RE was independent. The level of splicing reached under saturating conditions was linearly dependent on the number of *dsx*REs present (1–6, distributed along the exon 300–600 nts from the 3’SS), from which it was inferred that each *dsx*RE element was occupied and that the more that were occupied, the higher the chance of activation of splicing, as if the bound SR proteins each had a low probability of contacting a common target (20) (Figure 1A). This interpretation was substantiated by the use of tethered RS domains placed at various distances downstream of the 3’SS. The rates of splicing depended on the length of the RS domain, consistent with the existence of a common target, and declined with distance from the 3’SS as expected if the target were being found by looping (19). These results supported the models shown in Figure 1A and E.

Since then, the variety of sequences known to act in some contexts as ESEs or silencers has expanded considerably, following computational analyses (21,22), systematic screens of large sequence libraries (23,24) and the analysis of mutations (25–29). As a result, there is a much better understanding of the sequence preferences of the SR proteins, such as the prototypical SR protein, SRSF1 (30–34). Mapping of the transcriptome-wide binding sites by cross-linking *in vivo* confirms these preferences (34–37), and some analyses show binding sites enriched near splice sites. Despite this plethora of information, the limitations of all the widely used methods for studying splicing mechanisms have meant that there has been little progress in understanding the interactions of ESEs with trans-acting factors and the subsequent mechanisms of activation. The most informative experiments have come from the use of RS domains that were stably tethered to a 3′ ESE. These experiments showed that the RS domains contacted the branchpoint of the preceding intron (38,39) and stabilized U2 base-pairing (40). While these results support the idea of direct interactions or looping (Figure 1E), the use of tethering meant that the contribution of binding to the rate-determining step could not be assessed, the numbers of SR proteins bound were not known so propagation (Figure 1F) could not be excluded, and the contacts made were not, as had been expected, with U2AF proteins. Thus, the models inferred from the rates of *Drosophila* Tra/Tra2-dependent splicing are still generally used to represent or interpret mammalian ESE activity.

Doubts about the applicability of the *dsx*RE model to mammalian enhancers in general have arisen from analyses of complexes forming on ESEs. Using targeted oligonucleotide enhancers of splicing (TOES), we showed that splicing efficiency increased with the number of GGA repeat motifs in the enhancer domain of the oligonucleotide, consistent with the *dsx*RE results (41). However, extensive investigations using conventional methods showed that the enhancer sequence was able to form a number of different complexes, including a G-quadruplex, and that SR protein complexes under normal conditions were only minor components (42). This is consistent with other examples in which the proportion of cross-linking or affinity-purified proteins attributable to SR proteins in enhancer complexes has likewise been found to be very low (14,43), and suggested to us that SR proteins might interact only transiently with ESEs (42), as in the model in Figure 1D.

The best way to identify whether the limiting step in activation by an ESE is the binding of the SR protein or its subsequent interactions is to use multiple ESEs and test for multiple occupancy. Concurrent occupancy would indicate functionally stable binding and that, as with the dsxRE, subsequent interactions are limiting (Figure 1A–C). If multiple ESEs increased the rate of splicing but this was linked only to increased levels of single occupancy, then the probability of binding would be limiting (Figure 1D). The use of indirect methods, such as those used for the *dsx*RE, is inappropriate because the splicing rates do not saturate and it is difficult to infer much from a negative result (20). Direct measurement of the numbers of ESEs occupied would be a much more definitive approach. However, such measurements cannot be made in functional splicing assays for ESEs embedded in a pre-mRNA using conventional methods such as cross-linking, including CLIP, or affinity purification, because of the high levels of apparently non-specific association of SR proteins with RNA mediated by their RS domains, because SR proteins are recruited in other ways and because the distribution of pre-mRNA molecules among complexes with different numbers of molecules of protein bound is unknowable.

**Figure 2.**
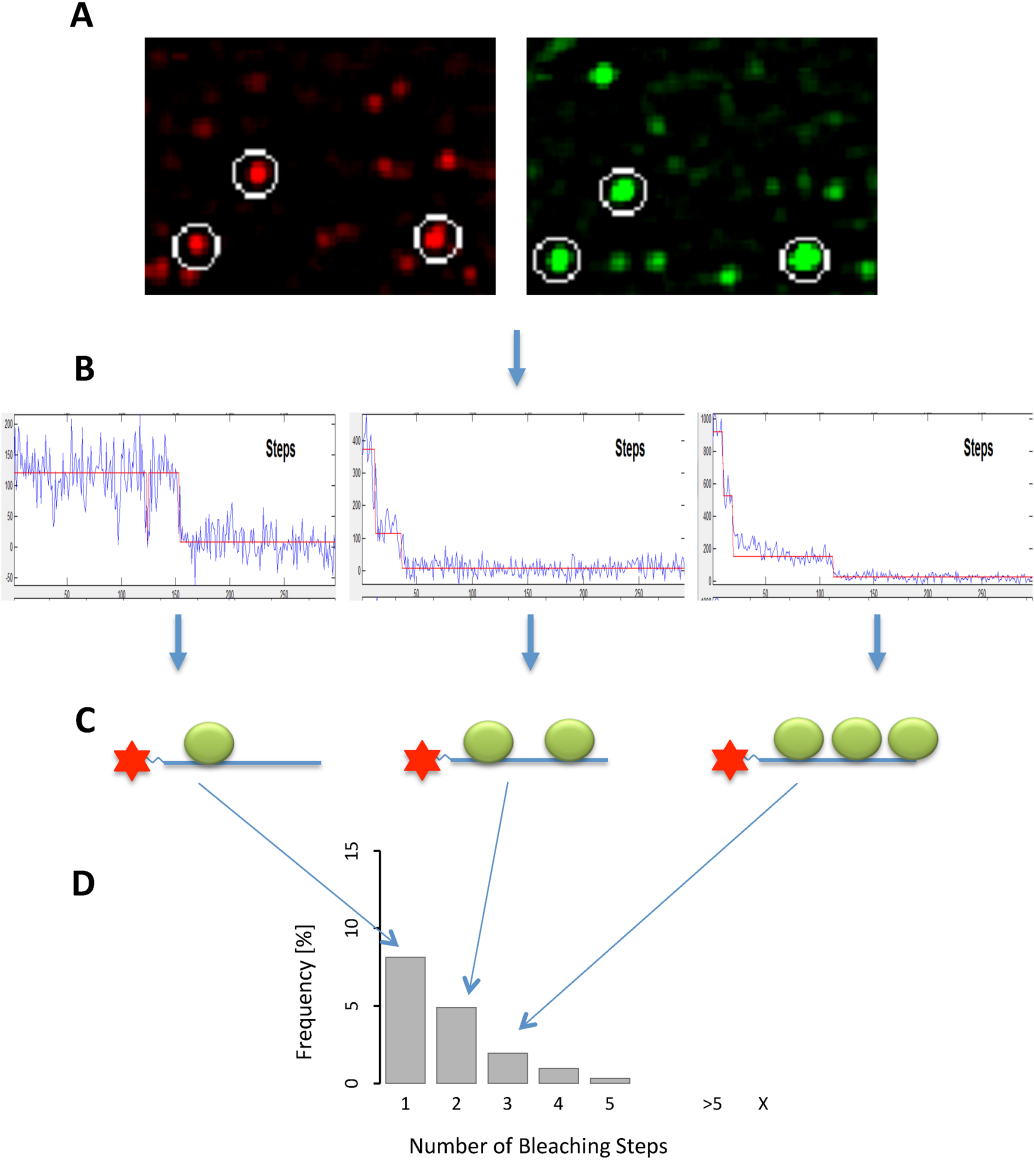
An outline of single molecule multicolour colocalization. (**A**) Representative views of a field of view showing the accumulated emission upon excitation at 640 nm (for RNA labelled with Cy5, red spots) and 488 nm (for mEGFP-SRSF1, green spots). Data was collected by emCCD from excitation at 640 nm for 50 frames of 50 ms and at 488 nm for 250 frames of 50 ms. Cy5-labelled RNA had been incubated in nuclear extract expressing mEGFP-SRSF1 under conditions allowing complex A formation before dilution and injection onto the slide surface for detection by total internal reflection fluorescence microscopy. Colocalized RNA and protein molecules are shown by circles. (**B**) Time courses of mEGP fluorescence from representative spots. Irreversible drops in the emission intensity are caused by bleaching of a single molecule of mEGFP. These are direct screen images and the ordinate (intensity per 50 ms frame) is re-scaled for each spot. (**C**) Diagrams showing for each molecule the number of mEGFP-SRSF1 molecules associated with the RNA, deduced from the bleaching profile. (**D**) The spots are classified and each contributes one RNA molecule to the classes represented in the histogram.

Single molecule multi-colour colocalization methods have been used extensively by this laboratory to determine directly the numbers of protein molecules bound to a molecule of pre-mRNA in mammalian nuclear extracts (44–47) (Figure 2) and by the laboratory of M. Moore to follow the dynamics of association and dissociation of splicing factors in yeast extracts (48,49), providing mechanistic evidence that would otherwise be unattainable. By combining single molecule methods with the use of non-RNA linkers, we describe a new paradigm for mammalian enhancers.

## MATERIALS AND METHODS

### Sequences

The transcripts comprised 225 nts of rabbit β-globin exon 2 and 67 nts of intron 2, fused to human SMN2 exon 7 and 157 nts of the preceding intron as described previously (42,50). In the control transcript, transcription terminated at nucleotide 48 in SMN2 exon 7, excluding the 6 nts preceding the 5′ splice site.

The ESE sequences appended to nt 48 were as follows:

ESE-A, CAAGGCGGAGGAAG;
ESE-B, CACACAGGACCACACAGGAC;
ESE-C, AAAAAGAAAGAAAAAAAGAAAGAA;
ESE-D, UCAGAGGAUCAGAGGA.

Tandem repeats of ESE-A were used as described.

In all the transcripts used for the assays with non-RNA linkers, the transcript lacked the Tra2β enhancer region (SMN2 exon 7 nts 17–28) and ended at nt 38. The removal of nts 17–28 shortened the exon by 12 nts, so that the addition of 12 abasic nts at the 3′ end followed by the ESE put the ESE at the same distance relative to the 3′ splice site as when the ESE was appended to the 3′ end as described above.

The transcripts used for splicing and cross-linking were prepared using T7 RNA polymerase with the cap analogue GpppG for initiation and [α-^32^P]GTP for elongation. The transcripts used for cross-linking were as follows, omitting the 5′ cap:

1 ESE, GCAAGGCGGAGGAAG;
2 ESEs, GCAAGGCGGAGGAAGCAAGGCGGAGGAAG;
3 ESEs, GCAAGGCGGAGGAAGCAAGGCGGAGGAAGCAAGGCGGAGGAAG;
4 ESEs, GCAAGGCGGAGGAAGCAAGGCGGAGGAAGCAAGGCGGAGGAAGCAAGGCG-GAGGAAG

These were purified by denaturing gel electrophoresis and quantified by scintillation counting.

### Synthesis and ligation of non-RNA linkers

The sequences ligated to nt 38 of SMN2 exon 7 were as follows:

UUCCUUAAAUCAAGGCGGAGGAAGCAAGGCGGAGGAAG (exon 7 nts 39–48 + ESE-Ax2).
UUCCUUAAAU – 2HEG – CAAGGCGGAGGAAGCAAGGCGGAGGAAG
UUCCUUAAAU – 12 abasic pd – CAAGGCGGAGGAAGCAAGGCGGAGGAAG
UUCCUUAAAU – 12 abasic pr – CAAGGCGGAGGAAGCAAGGCGGAGGAAG

The synthesis of the linkers is described in detail elsewhere (Reichenbach *et al.*, ms in preparation). In brief, TOM-Protected RNA phosphoramidites, HEG spacer phosphoramidite and CPG supports loaded with standard nucleosides were purchased from LINK Technologies Ltd (Bellshill, UK) and Cambio Ltd. (Cambridge, UK). Abasic phosphoramidites were synthesized based on methods reported in the literature (51,52). RNA oligonucleotides were synthesized using standard solid phase oligonucleotide synthesis protocols on an ABI 394 synthesizer. The reaction time for each individual coupling was 10 min. The crude oligonucleotides were purified by gel electrophoresis before ligation to the transcripts with 20 nt DNA oligonucleotide splints and T4 RNA ligase 2 (53).

### Labelling RNA at the 5′ end

Substrate RNAs for single molecule detection were transcribed in the presence of 10 mM guanosine-5′-*O*-monophosphorothioate (BioLog), and the 5′ end was labelled using 5′ Cy5-maleimide (54), as described (47).

### Splicing and analysis of complexes

The HeLa cell nuclear extract containing mEGFP-SRSF1 and mCherry-U1A was prepared (55) and the relative levels of exogenous and endogenous proteins were characterized by western blotting and detection with a fluorescent secondary antibodies (IRDye 680LT, LI-COR 926–68020; IRDye 800CW, LI-COR 926–32211). Splicing assays and native gel electrophoresis of splicing complexes were done as reported previously (45). Splicing levels were taken as the molecular ratio of mRNA/(mRNA + pre-mRNA), after allowing for the number of radioactive nucleotides in each species. For cross-linking reactions, the ESE transcripts were incubated in 10 μl in splicing conditions for 15 min, then irradiated with a short-wave SpotCure (UVP) for 2 min and incubated with 2 μg RNase A and 5 U RNase T1 before electrophoresis and transfer onto nitrocellulose.

### Sample preparation

Cover slips from Menzel-Gläser (22 mm × 50 mm, #1) were soaked in 1 M KOH for four hours before being washed with water, sonicated, dried under a nitrogen stream and cleaned in an argon plasma (MiniFlecto-PC-MFC, Gala Instruments) for 5 times 5 min. Sample chambers were formed with double-sided tape and a second cover slip. Splicing reactions were prepared with 50% nuclear extract, 3.2 mM MgCl_2_, 50 mM monopotassium glutamate, 20 mM phosphocreatine, 1.5 mM ATP, 20 mM Hepes pH 7.5 and 3 units RNase OUT (Invitrogen). A 2′-*O*-methyl oligonucleotide complementary to U6 snRNA was added at 1 μM to block splicing at complex A (45,56). The reaction mixtures were pre-incubated for 15 min at 30°C before pre-mRNA was added at a final concentration of 62.5 nM and incubated for a further 15 min at 30°C. Samples were diluted in 40 mM Hepes pH 7.5, 3.2 mM MgCl_2_, 50 mM monopotassium glutamate, 50 mM KCl, 0.1 mM EDTA and 0.5 mM DTT, loaded into the sample chamber and incubated for 5 min.

### Data acquisition and analysis

A home-built objective-based total internal reflection fluorescence microscope was used for the single-molecule experiments. The central regions of both the laser beam and the CCD chip were used to reduce intensity variation. A stable focus was achieved by active feedback from the reflected beam. Nine fields were acquired in succession automatically, and from each experiment ∼50 fields in total were acquired. Each field was obtained by collection of 50 frames, each of 50 ms, with excitation at 633 nm, followed by the collection of 250 frames with excitation at 488 nm. The periods of irradiation resulted in the complete bleaching of Cy5 (pre-mRNA) and mEGFP in each case. Recording was continuous, minimizing the possibility of unrecorded bleaching of mEGFP during the switch between lasers. Each SRSF1 experiment was done on three separate occasions.

### Data analysis

The data were analyzed using a MATLAB program. Spots produced by fluorescence were identified by detecting squares in which the maximum pixel intensity exceeded the mean by a threshold value, tested for the presence of only a single peak, and required to conform to a Gaussian distribution in each dimension. The use of maximum intensities during the recording period ensures that mEGFP molecules that were in a dark state at any point, even repeatedly, would be detected during the 12.5 s of irradiation at 488 nm because the dark state lifetime of mEGFP is only 1–2 s (57–59). Chromatic aberrations were corrected by a linear transformation, using parameters from a test molecule labelled with two fluorophores. Steps in the bleaching profile were identified using a recursive Bayesian strategy (Jobbins *et al.*, submitted). Each frequency histogram shows the result of measurements collated from around 150 fields (three experiments done on separate days, each comprising 50 fields). The colocalization values represent the mean from the three experiments, with the standard error of the mean. Apparent colocalization arising from random coincidence was calculated for each image and would involve 1–2% of the pre-mRNA molecules. This was considered to be insignificant and was not subtracted from the values measured. The frequency values are the total from the three experiments, with error bars showing the square root of the variance of the binomial probability that an RNA spot will be associated with the given number of protein bleaching steps.

The distribution of frequencies corresponding to the presence of a single molecule of SRSF1 in a complex was taken to be the same as was seen under the same conditions but in the absence of pre-mRNA (Supplementary Figure S1). The level of dimerization of the protein was calculated from the proportion of complexes observed to contain two molecules of mEGFP-SRSF1 (12%). The distributions expected from two molecules in a complex (Supplementary Figure S1) were calculated from the proportion of SRSF1 in the extract that was labelled with mEGFP (58%) and the level of dimerization (45,47). The level of mis-folding of the mEGFP domain was assumed to be nil, both because it was N-terminal to the SRSF1 moiety and because the results in Figure 5 fit the prediction for binding by two molecules of SRSF1 in 100% of the complexes with no indication of the presence of larger complexes.

## RESULTS

### The dependence of splicing on ESE sequences is linked to the level of ESE-dependent binding by SRSF1

**Figure 3.**
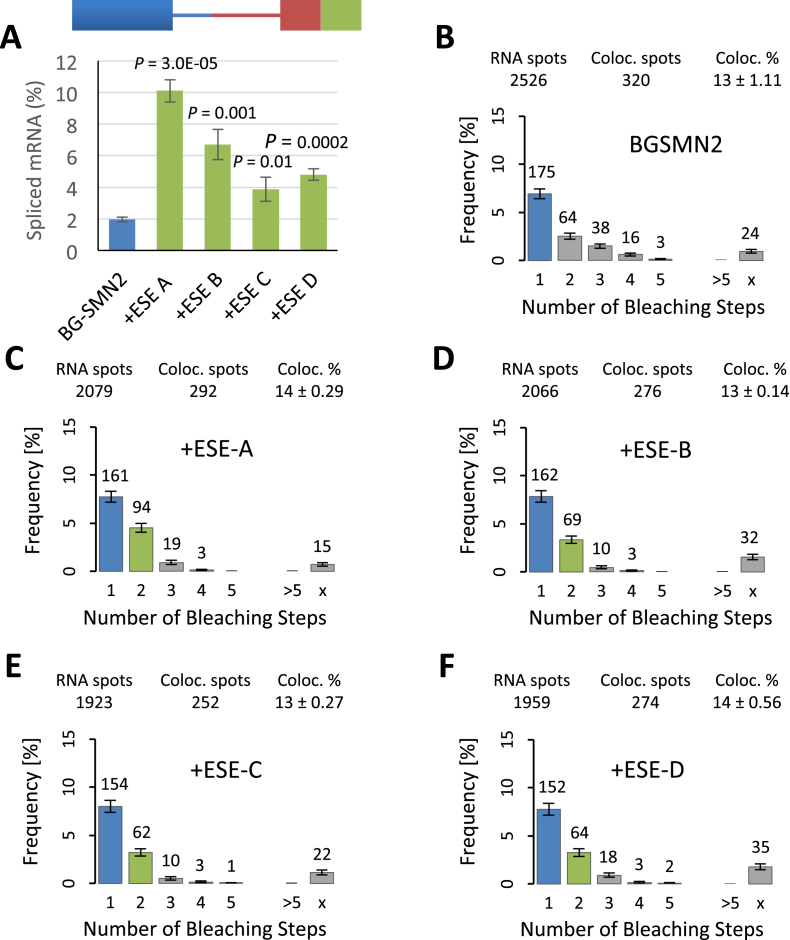
Effects of different ESE sequences on splicing and the association of SRSF1. (**A**) Splicing activity *in vitro* of pre-mRNA with different 3’ESEs. The pre-mRNA is represented by a diagram showing the sequences from β-globin exon 2 (blue), SMN2 exon 7 (brown) and the ESE under test (green). The level of spliced mRNA (%, [mRNA]/[mRNA + pre-mRNA]) after incubation for 2 h was calculated for each of three reactions done in triplicate, and the mean and standard error of the mean are shown. The probabilities (*P*) that results with the ESE-containing reactions are from the same population as the results from the pre-mRNA lacking an extra ESE (BGSMN2) were calculated by a Student's *t* test. (**B**) Single molecule multicolour colocalization studies on the levels and stoichiometries of mEGFP-SRSF1 binding to molecules of labelled pre-mRNA in nuclear extracts. Histograms show the frequencies (%) of pre-mRNA (BGSMN2) molecules showing bleaching of colocalized labelled protein in **n** steps (Figure 2). The number above each bar indicates the number of complexes in which complete bleaching was achieved in 1, 2, 3, etc., steps, and hence the number of complexes in which there were 1, 2, 3, etc., molecules of fluorescent fusion protein. > refers to complexes where more than 5 bleaching steps were measured; x represents complexes where the number could not be determined. The percentage value above each histogram is the percentage of labelled pre-mRNA molecules (RNA spots) that were associated with mEGFP-SRSF1 (Coloc. spots). Nuclear extracts contained ATP and an oligonucleotide complementary to U6 snRNA that blocks progression beyond complex A. (**C**) Frequency distributions as described above for pre-mRNA comprising BGSMN2 with a 3’ESE containing one motif of ESE-A from Ron exon 12 (60,61). The frequency for *n* = 1 is blue and that for *n* = 2 is green to assist in identifying these classes (see text). (**D**) As (C), but the pre-mRNA contained two 3′-terminal repeats of an SRSF1 site of action determined by functional SELEX (62). (**E**) As (C), but with two 3′ terminal repeats of the SMN2 exon 7 Tra2 β binding site (63,64). (**F**) As (C), but with two 3′ terminal repeats of an SRSF1 site of action inferred from RNA-seq (34).

To test whether there was a correlation between the efficacy of an ESE and binding of SRSF1, four different sequences were tested: ESE-A, the known SRSF1 binding site from Ron exon 12 (60,61); ESE-B, an SRSF1 binding motif identified by functional SELEX (62); ESE-C, the Tra2β binding site from SMN exon 7, which has been shown to promote splicing but should not bind SRSF1 (63,64); ESE-D, an SRSF1 motif established via RNA-seq (34). Since ESE-A was longer than the others (14 nt), the others were incorporated as tandem repeats with overall lengths of 20, 24 and 16 nt, respectively. Each ESE was attached to the 3′ end of SMN2 exon 7 in a β-globin/SMN2 chimeric pre-mRNA (47). In vitro splicing assays showed that all four ESEs increased the level of splicing significantly, but the ESE derived from Ron exon 12 was by far the most effective (Figure 3A).

Transcripts labelled with Cy5 were incubated in nuclear extract prepared from cells expressing functional mEGFP-SRSF1 (Jobbins *et al.*, submitted), in the presence of a 2′-*O*-methyl oligonucleotide complementary to U6 snRNA that causes spliceosome assembly to stall at complex A (45,56). The reactions were then diluted and captured on cover slips. The fluorescent RNA and proteins were imaged using TIRF at two or three wavelengths and fluorescence was recorded until the fluorophores were bleached. Co-localized single molecules of RNA with either protein were identified and the number of steps in which the mEGFP or mCherry signal bleached was recorded for each RNA molecule (Figure 2). The numbers of spots in which bleaching occurred in 1, 2, 3 etc. steps are shown as a percentage of the total number of RNA spots (Figure 3B–F). Each distribution is an accumulation of the spots from three separate experiments.

In the absence of an ESE, there was a strong peak of complexes containing a single molecule of mEGFP-SRSF1, followed by a geometric distribution of higher order complexes (Figure 3B, BGSMN2; *P_geo(n = 2–5)_* = 0.15). We have shown elsewhere that U1 snRNPs recruit a single SRSF1 to a 5’SS in complex A but that failure to form complex A leads to a geometric distribution associated with non-productive complexes (Jobbins *et al.*, submitted). We conclude that only a minority of BGSMN2 pre-mRNA had assembled into complex A, while the majority had not (see below). The addition of the ESEs reduced the levels of the non-productive complexes with 3 or 4 molecules of mEGFP-SRSF1 and produced an increase in the proportion with two molecules (Figure 3C–F). Supplementary Figure S1 shows the frequency distributions expected if all the complexes contained either one or two molecules of SRSF1, taking into account the proportions of labelled and unlabelled SRSF1 in the extract and the levels of dimerization observed in the absence of pre-mRNA. If all the colocalized complexes contained two molecules of SRSF1 then, as shown in Supplementary Figure S1, the number of molecules of RNA in complexes that bleached in two steps would be ∼90% of the number bleaching in one step. The ratio observed in the absence of an ESE, when many of the molecules are in non-productive complexes, is 37% (Figure 3B), but it increases to 58% with ESE-A (Figure 3C) and is around 40–43% with the other ESEs (Figure 3D–F) We conclude that the presence of an ESE reduces the proportion of non-productive complexes and increases the proportion of complexes containing two molecules of SRSF1. Significantly, the abundance of these complexes was highest with the most active ESE, ESE-A, consistent with the expectation that the efficiency of an ESE is linked to its ability to bind SRSF1. We have shown elsewhere that inclusion of an oligonucleotide complementary to the 5′ end of U1 snRNA eliminates the second peak with ESE-A, demonstrating that recruitment by U1 snRNP and the ESE are independent (Jobbins *et al.*, submitted).

### Multiple ESEs produce additive effects on splicing but do not increase the number of SRSF1 molecules bound

**Figure 4.**
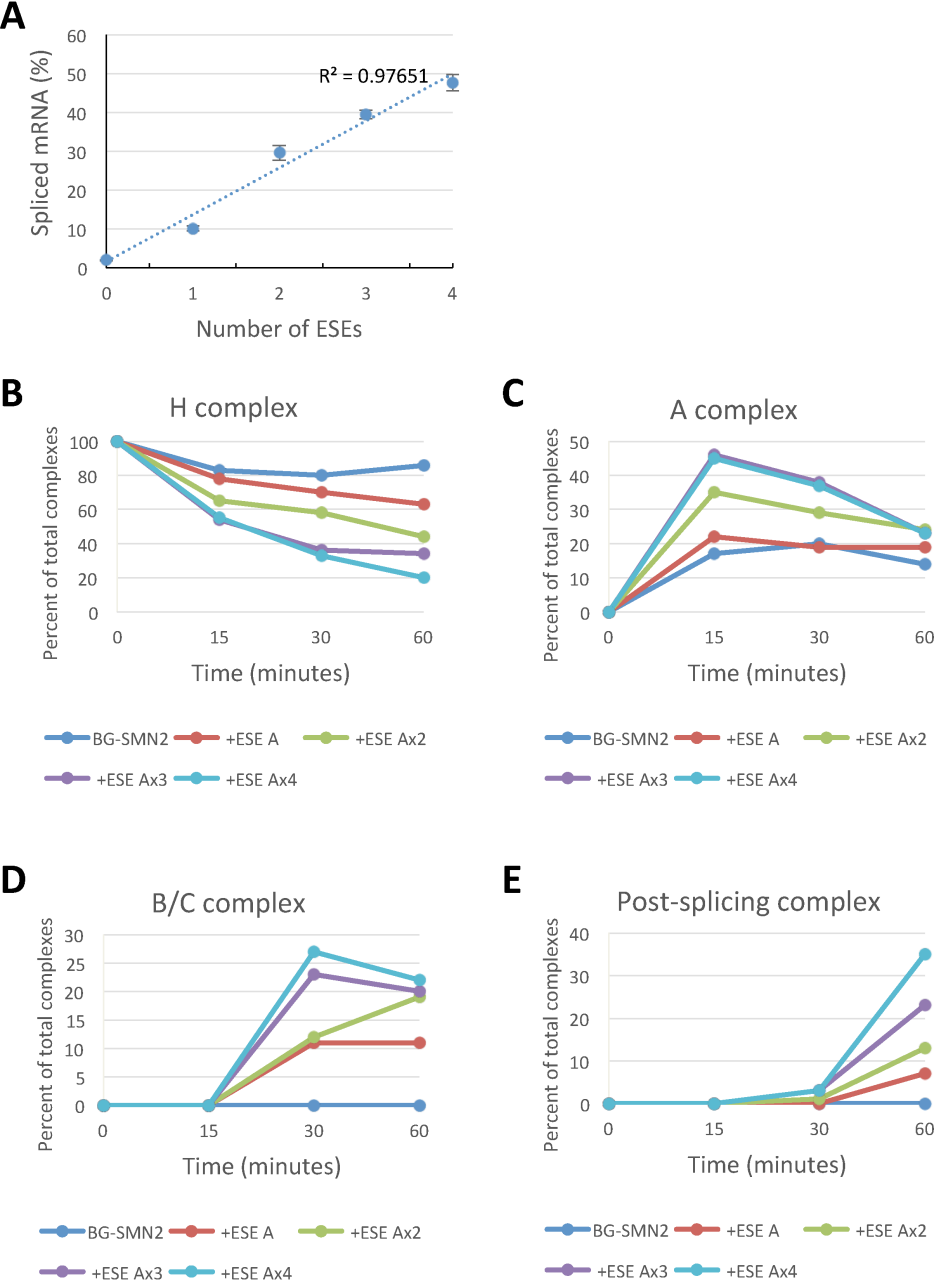
Effects of multiple copies of the Ron ESE-A sequence on splicing and complex assembly. (**A**) The level of spliced mRNA after incubation for two hours with BGSMN2 pre-mRNA terminating in 0, 1, 2, 3 or 4 copies of ESE-A. (**B**–**E**) Relative intensities of complexes containing labelled RNA after analysis by native gel electrophoresis of splicing reactions containing the indicated pre-mRNA that had been incubated for the times shown.

Introducing tandem repeats of ESE-A produced an increase in the level of splicing that was proportional to the number of ESEs (Figure 4A, *R*^2^ = 0.98; Supplementary Figure S2A), very like that seen with *dsx* (20) or TOES oligonucleotides (42). There was a corresponding increase in the rates and levels of formation of splicing complexes at all stages (Figure 4B–E; Supplementary Figure S2B).

The observation of linearity is a fundamental starting point in assessing possible pathways for ESE action. It is clearly consistent with either the *dsx* mechanism (Figure 1A), if each additional protein bound were to add to a very small probability of an interaction, but it might also be explained by other mechanisms such as those in Figure 1B, involving multiple targets if each target contacted has an approximately equal effect on the rate, Figure 1C, in which binding is cooperative but each additional site occupied contributes progressively less to the reduction in ΔG, or Figure 1D, in which the probability of binding by a protein is very low but each additional site contributes independently.

**Figure 5.**
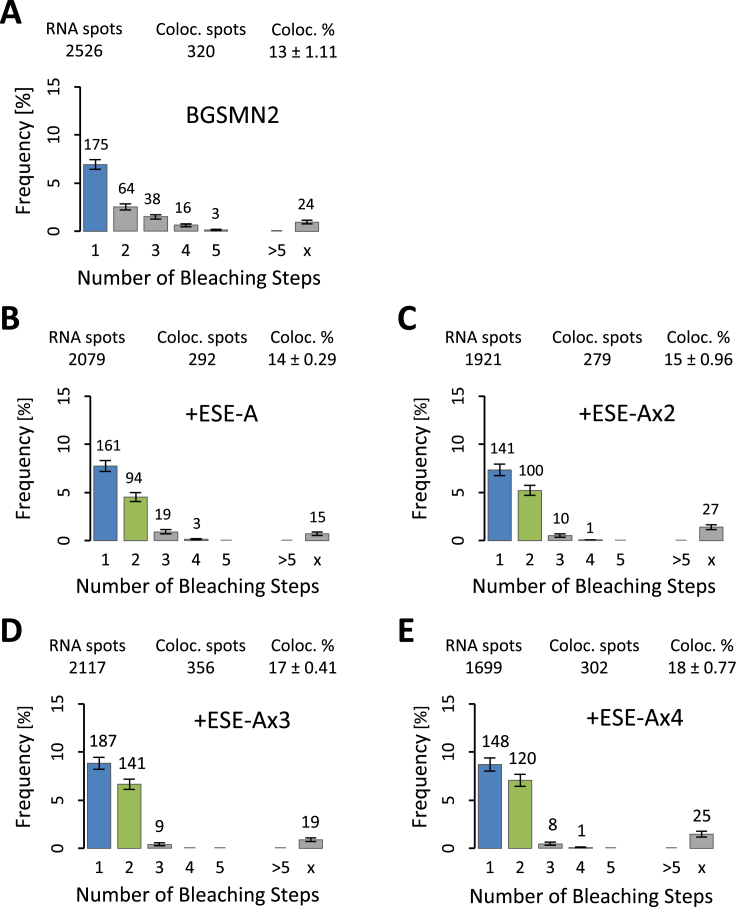
Single molecule multicolour colocalization studies on the levels and stoichiometries of mEGFP-SRSF1 binding in nuclear extracts to molecules of BGSMN2 pre-mRNA terminating in 0, 1, 2, 3 or 4 copies of ESE-A. As in Figure 3, histograms show the frequencies (%) of pre-mRNA molecules showing bleaching of colocalized labelled protein in **n** steps, and the overall proportion of pre-mRNA showing colocalization is shown at the top. (**A**–**E**), pre-mRNA terminating in 0, 1, 2, 3 or 4 copies of ESE-A. Panels A and B are reproduced from Figure 3B and C.

**Figure 6.**
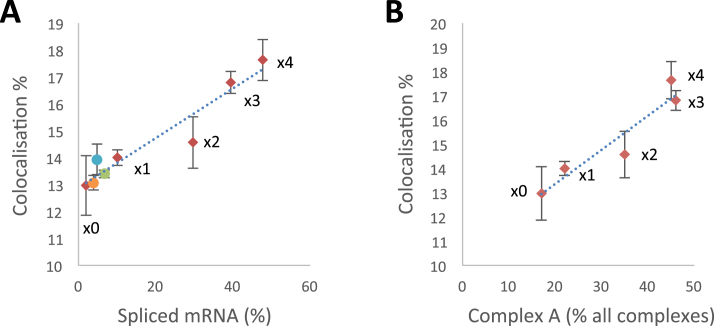
The total proportion of pre-mRNA molecules colocalized with molecules of mEGFP-SRSF1 (from all the experiments shown in Figures 3 and 5) compared with the efficiencies of splicing (**A**; data from Figures 3A and 4A) or complex A formation (**B**; data from Figure 4C, 15 min time point). Points denoted by red diamonds were obtained with BGSMN2 with 0, 1, 2, 3 or 4 copies of ESE-A, as marked; the results with ESE-B, ESE-C and ESE-D are shown as green, orange and blue circles, respectively.

The extent of multiple occupancy was measured for each pre-mRNA as above. The results (Figure 5) showed that the increase in the number of ESEs was accompanied by a small increase in the levels of colocalization, i.e. the proportion of pre-mRNA molecules bound. There was a roughly linear relationship between the level of co-localization and the levels of both splicing and complex A formation (Figure 6; A, *R*^2^ = 0.92 and *P*_correlation_ < 0.001; B, *R*^2^ = 0.89 and *P*_no correlation_ < 0.05), but in both cases the intercept on the ordinate is positive due to the binding of mEGFP-SRSF1 via U1 snRNPs and background binding.

The most striking feature of Figure 5 is that the increase in the number of ESEs produced no increase at all in binding of three, four or five molecules of SRSF1, although there was an increase in the proportion of pre-mRNA molecules associated with two molecules of mEGFP-SRSF1. Specifically, the number of complexes in which mEGFP bleached in two steps compared to those in which it bleached in one step rose from 58% to 71%, 75% and 81% as the number of ESEs increased from one to four. Since the increased number of ESEs produced a clear functional effect on splicing, it is unlikely that the apparent restriction of stable binding to one molecule of SRSF1 results from steric occlusion of the extra copies of the ESE by, for example, G-quadruplex formation. However, we tested this possibility by UV cross-linking with the ESE sequences. If only a single molecule of SRSF1 were able to interact with the tandemly-repeated ESEs, then the addition of an equal number of molecules with 1, 2 3 or 4 ESEs to nuclear extract would produce equal levels of cross-linking. However, the results showed a linear increase in cross-linking to SRSF1 and other proteins as the number of repeats increased (Supplementary Figure S3), meaning that there is no intrinsic barrier to protein interactions with the tandem repeats.

We conclude that the affinity of SRSF1 for an ESE is low and that the probability of binding SRSF1 is rate-limiting, as illustrated in Figure 1D. The concurrent loss of background binding and the native gel results support the possibility that binding of the second SRSF1 strengthens the 3’SS, stimulating complex A formation. Finally, we can conclude that the activation step does not involve propagation of SRSF1 (Figure 1F), although it is possible that other proteins might be involved.

### ESE-A increases the association of U2AF35 and U2 snRNP

To confirm that the ESE repeats affect the recruitment of 3’SS components, the Cy5-labelled BGSMN2 and BGSMN2 + ESE-Ax4 pre-mRNAs were incubated in complex A-forming conditions in nuclear extracts containing either mCherry-U2AF35 + mEGFP-U2AF65 or mEGFP-U2B’’ (47). The results in Supplementary Figure S4 show that the four copies of ESE-A produced a marked (about two-fold) increase in the co-localization of U2AF and U2 snRNPs with pre-mRNA. We conclude that the single molecule results are consistent with the results of conventional experiments.

### A Strong ESE functions by looping to its target site

The results described above are consistent with activation via looping, although other mechanisms are possible, such as propagation of other proteins from SRSF1 or even some form of sliding or reeling of a protein complex relative to the RNA. To test these, we introduced non-RNA linkers between the 3’SS and ESE-Ax2 (Reichenbach *et al.*, manuscript in preparation). The linkers comprised tandem repeats of hexaethyleneglycol (HEG) or 12 (deoxy)ribose-phosphodiester linkages of abasic DNA or abasic RNA, which were intended to provide flexibility while disrupting the interactions of RNA-binding proteins that might propagate or slide from SRSF1. Each linker was prepared by solid phase synthesis with a portion of the 3′ exon of SMN2 exon 7 at the 5′ end and two copies of ESE-A at the 3′ end. The linkers were attached by ligation to the body of BGSMN2. The distance from the 3’SS to the ESE was maintained by deleting 12 nts from the exon. The sequences removed contained the Tra2β enhancer region, which is superfluous in the presence of an additional SRSF1-dependent enhancer (42) (*P*_same population_ = 0.12; Supplementary Figure S5). The 2 × HEG linkers would be expected to be at least as flexible as the RNA sequence, since their length of ∼4.4 nm is about eight times the persistence length (65,66) and an unfolded RNA chain of 12 nts would have a contour length of up to ∼7 nm, which is about 3.4 persistence lengths (67).

**Figure 7.**
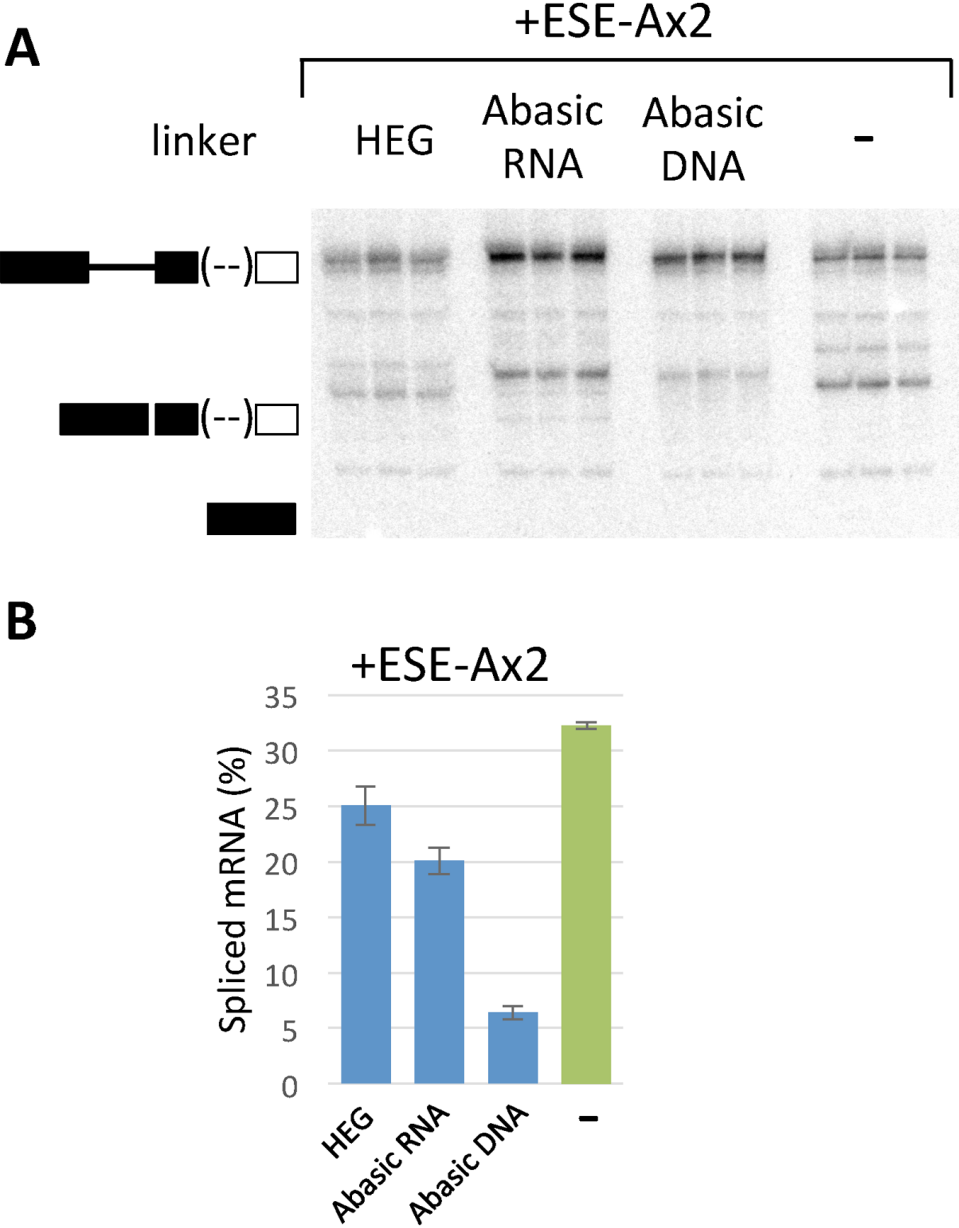
Effects of introducing non-RNA linkers between the 3’SS and the ESE or a 5’SS. (**A**) *In vitro* splicing reactions done in triplicate for 2 h with pre-mRNA containing hexaethyleneglycol, abasic RNA or abasic DNA linkers, or without a linker. In all cases, two copies of the ESE-A motif were attached at the 3′ terminus. (**B**) Quantitative results from the experiment in (A). The histogram shows the mean values and error bars show the S.E.M.

Splicing assays showed that the abasic RNA linker and HEG linkers did not block the ESE, even though its activity was reduced (Figure 7A and B). In contrast, the abasic DNA linker reduced splicing to the level seen in the absence of an ESE. Mass spectrometric analysis of proteins bound to the linkers in nuclear extract showed that the abasic DNA could bind known DNA damage repair proteins (manuscript in preparation). It is possible that the recruitment of these non-splicing factors interferes with ESE activity.

We conclude from the ability of the ESE to act across a bridge of abasic RNA or HEG, without bases or even phosphodiester linkages, that the bound SR protein is most likely to act by looping (Figure 1E) rather than propagation of protein complexes along the RNA or by sliding.

## DISCUSSION

The results described here show that tandemly repeated ESEs increased splicing efficiency linearly, but that only a single molecule of SRSF1 was recruited by the ESEs in addition to the one recruited by the 5’SS-bound U1 snRNP. The activity of the ESEs was not blocked by the introduction of non-RNA linkers between the 3’SS and the ESE. The best explanation for these observations is that SRSF1 binds very weakly, associating only transiently with an ESE, but that increasing the number of ESE repeats increases the probability that a molecule of SRSF1 bound to an ESE will interact with its target 3’SS (Figure 1D). It is this molecule of SRSF1 that is then bound stably and detected in our experiments. The interaction step is likely to be direct, involving 3D diffusion and collision (Figure 1E).

The results shown here demonstrate the reasons why conventional, ensemble methods could not be used to test the hypotheses described. There is a high level of apparently non-specific binding that follows a geometrical distribution (e.g. Figure 3B), and the addition of ESEs has a relatively small effect on the proportion of RNA bound by SRSF1; instead, the distribution of numbers of molecules of SRSF1 on a molecule of pre-mRNA changes, and the geometrical distribution shrinks in importance. On pre-mRNA with a strong 3’SS, a similar geometric distribution is seen when U1 binding is prevented (Jobbins *et al.*, submitted). Thus, single molecule measurements of binding stoichiometry enable novel insights. One of the limitations in our results is that we cannot determine the exact proportion of complexes associated with two molecules of SRF1. This is the result of an under-representation of complexes bleaching in three steps, which should arise from the intrinsic dimerization of mEGFP-SRSF1 when two molecules of SRSF1 are bound (c.f. Supplementary Figure S1). It is possible that the interactions resulting in dimerization are occluded in complex A. This limitation does not, however, affect the obvious conclusions that the proportions of complexes containing two molecules of SRSF1 depend on the identity of the ESE and increase with the number of ESE repeats.

One difficulty with single molecule methods is that their most radical findings of necessity cannot be confirmed by conventional methods (48). However, we have repeatedly demonstrated that our results fit those obtained previously by ensemble methods where the strategies intersect. Examples include: our discovery that 3 molecules of PTB are bound to either side of the repressed exon 3 of *Tpm1* matched the number of potential binding sites for this multi-domain protein (44); the demonstration that PTB binding sites antagonised U2AF65 binding in *Tpm1* (47); the numbers of U1 snRNPs bound to alternative 5′ splice sites were exactly in agreement with our earlier ribonuclease H protection assays showing that both alternative sites could be occupied even when only one site was used (45); the finding that in a complete three-exon transcript the difference in sequence between exon 7 of SMN1 and SMN2 produced a major effect on U2 snRNP binding (47); the demonstration that an SRSF1-dependent ESE bound SRSF1 (this work); the observation that ESEs that stimulated splicing and complex A formation also stimulated the recruitment of a U2 snRNP (this work). Thus, while ensemble methods may not be able to directly validate single molecule results, the fact that single molecule results can replicate the results of ensemble methods, often with better precision, supports the validity of the unexpected findings too.

The weakness of the initial interactions of SRSF1 with the ESE suggests that the protein binds independently, rather than as part of a complex such as the Tra/Tra2/SR complex that binds *dsx* repeats. The Kd of SRSF1 (lacking the RS domain) for an optimal site was ∼0.2 μM (34), and in functional conditions competition with other proteins may reduce the rate of binding or even facilitate dissociation. The transience of such interactions has interesting implications. One is that, prior to the interactions between a bound SR protein and 3’SS components, it seems improbable that stable or stoichiometrically defined complexes will form on an exon. If other proteins behave like SRSF1, then the multiple proteins associating with an exon are likely to do so dynamically, binding in numerous combinations over short periods of time. This would resolve the dilemma that more proteins appear to be able to bind to SMN2 exon 7 and affect its splicing than could possibly bind concurrently (68). On the other hand, this would complicate our ability to determine which sets of proteins are consistent with activation.

The other question that arises is in regard to cross-linking assays, including transcriptome-wide CLIP. These will naturally tend to detect SRSF1 stabilized by post-binding interactions rather than molecules engaged in transient initial binding to the RNA. Thus, if multiple ESEs contribute towards an outcome, then it is possible that only one ESE would be bound stably on each transcript and it would be by far the most likely to be detected. The overall signal for that event for many molecules would be spread among all the contributing ESEs, and the apparent significance of the ESEs is likely to be under-estimated. This might have contributed to the difficulty of identifying the connection between the enrichment of SRSF1 binding sites in the transcriptome near 3’SS and the likelihood of skipping or inclusion (34,37).

The use of non-RNA linkers to differentiate between direct through-space interactions and processive processes (propagation or scanning) in splicing was applied first to determine the basis of the interaction between splice sites across an intron (69). We used the strategy subsequently to investigate the mechanisms of action of ESEs in a trans-acting TOES oligonucleotide that stimulated inclusion of SMN2 exon 7 in a three-exon pre-mRNA (70). Like the ESE investigated here, it recruited SRSF1 and stimulated the recruitment of U2 snRNP and associated proteins (42). However, the hexaethyleneglycol linker was inserted via copper-catalyzed alkyne-azide cycloaddition (CuAAC) ligation, forming exclusively a 1,4-triazole between the annealing domain and the ESE domain. The activity of the TOES oligonucleotide was actually increased by the inclusion of one or two units of hexaethylene glycol, but this improvement was lost if ten, eleven or twenty units were included, which is consistent with interactions by diffusion (70). The work described here with 3’ESE sequences that act in cis confirms that intervening non-RNA linkers do not prevent the activation of a 3’SS by an ESE. Our conclusion that the activation step involves looping is in agreement with that of Shen & Green (2004), who used an MS2-tethered RS domain with an intervening TEV cleavage site to show that the RS domain tethered to the 3′ exon could be cross-linked to the branch site in complex A (38).

A different outcome was found when we tested the effects of a non-RNA linker on a 5’ESE that affects 5’SS selection (71). The activity of the 5’ESE was ablated by inclusion of hexaethyleneglycol, inserted via CuAAC ligation between the ESE and the exon upstream of the target 5’SS. It is hard to resist the inference that the effects of an ESE on a 5′ splice site are mediated by a process involving contact with the intervening RNA, and thus are quite different from the looping process in which an ESE activates a 3’SS. The possibility that these are quite different processes is in agreement with the observations that the ability of SRSF1 to stimulate U1 snRNP binding to a 5’SS does not require the RS domain (61,72,73), whereas the tethering experiments have shown that the activation step for 3’SSs requires only the RS domain (19,38). It remains to be seen whether such differences are characteristic of the mechanisms by which ESEs affect 5’SSs as opposed to 3’SSs or whether there are other important determinants of the activation step mechanism.

The picture that emerges from these experiments is that SRSF1 binds transiently to SRSF1-dependent ESEs, and that each ESE makes an independent but effectively additive contribution to the probability that one SRSF1 molecule will be locked into place in complex A by interactions with other components of the early splicing complexes. It is not clear yet to what extent this new model applies to the many other proteins that interact with exons. It has been estimated that most positions in a short exon contribute significantly to the outcome of splicing (25,27,74) and that mutations act independently and additively (74,75). This is consistent with the possibility that most interacting proteins bind transiently (74), as we have inferred for SRSF1, and that the exon–protein complex is in a state of rapid flux. If so, it may be that none of the many possible combinations of proteins bound to the exon is uniquely required for activation. If repressor proteins behave similarly and are purely competitive antagonists, then even an exon with a heavy preponderance of silencers would be bound eventually by an SR protein and make contact with the 3′ splice site. To prevent this, there may be a time limit, creating a window of opportunity within which an ESE-bound SR protein might contact the 3′ splice site. The limit might be imposed by the slower formation of stable repressor complexes, such as those of PTBP1 around *Tpm1* exon 3 (44) or exon N1 of Src (76), or of Rbfox in LASR complexes (77), or more generally by a transition into an alternative pathway, both *in vivo* and *in vitro*. The existence and nature of any limiting events have yet to be determined.

## Supplementary Material

Supplementary Data
