# Supplementary material for: The mechanisms of a mammalian splicing enhancer
Source: Nucleic Acids Res. 2018 Jan 31;46(5):2145–58. doi: 10.1093/nar/gky056 (PMC5861446; doi:10.1093/nar/gky056)
Supplement: Supplementary Data [file gky056_supp.pdf]

Supplementary Material

The mechanisms of a mammalian splicing enhancer

Andrew M. Jobbins[1†], Linus F. Reichenbach[2†], Christian M. Lucas[1], Andrew J. Hudson[3], Glenn A. Burley[2*] and Ian C. Eperon[1*]

[1]Leicester Institute of Structural & Chemical Biology and Department of Molecular & Cell Biology, University of Leicester, UK

[2]Department of Pure and Applied Chemistry, University of Strathclyde, UK

[3]Leicester Institute of Structural & Chemical Biology and Department of Chemistry, University of Leicester, UK.

Supplementary Figure Figure S1

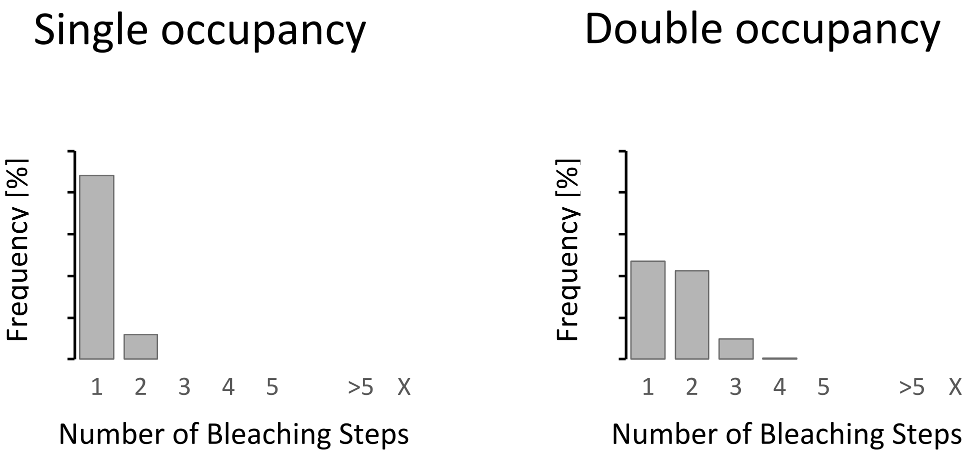

Supplementary Figure Figure S2

A

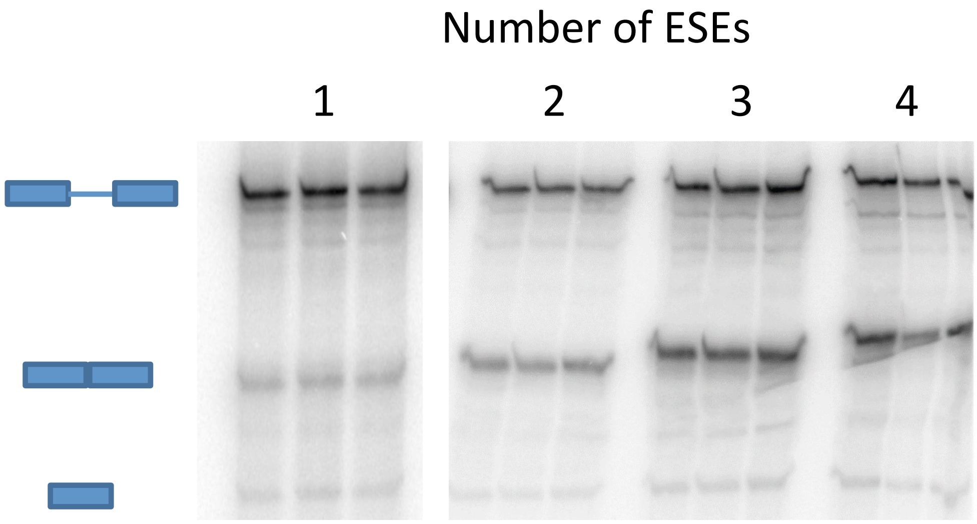

B

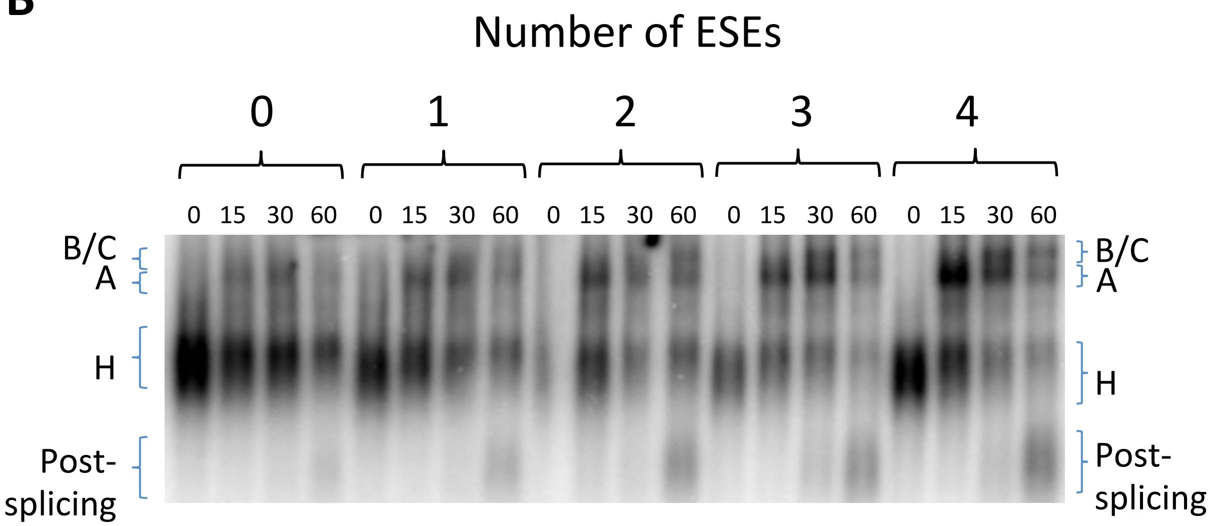

Supplementary Figure Figure S3

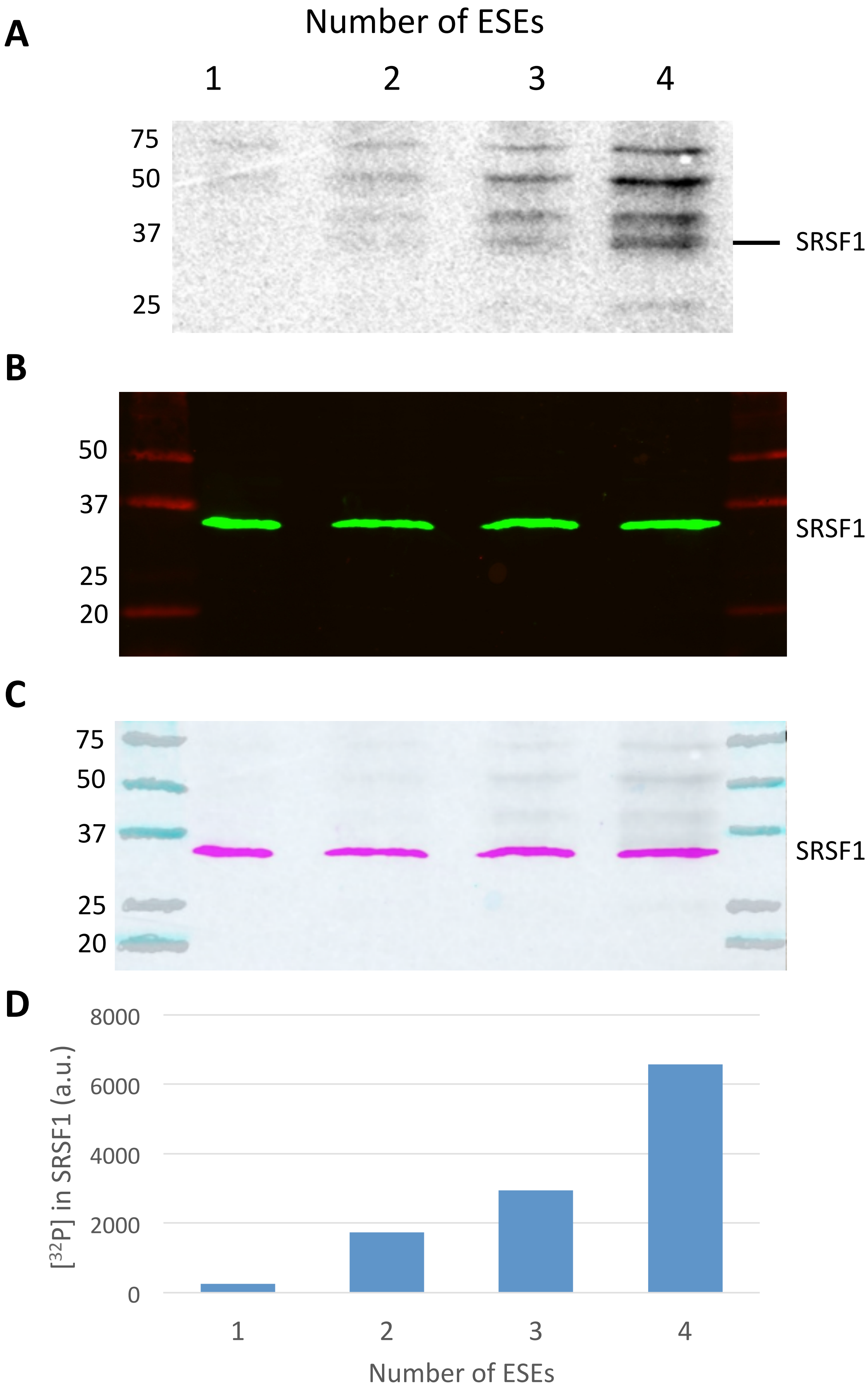

Supplementary Figure Figure S4

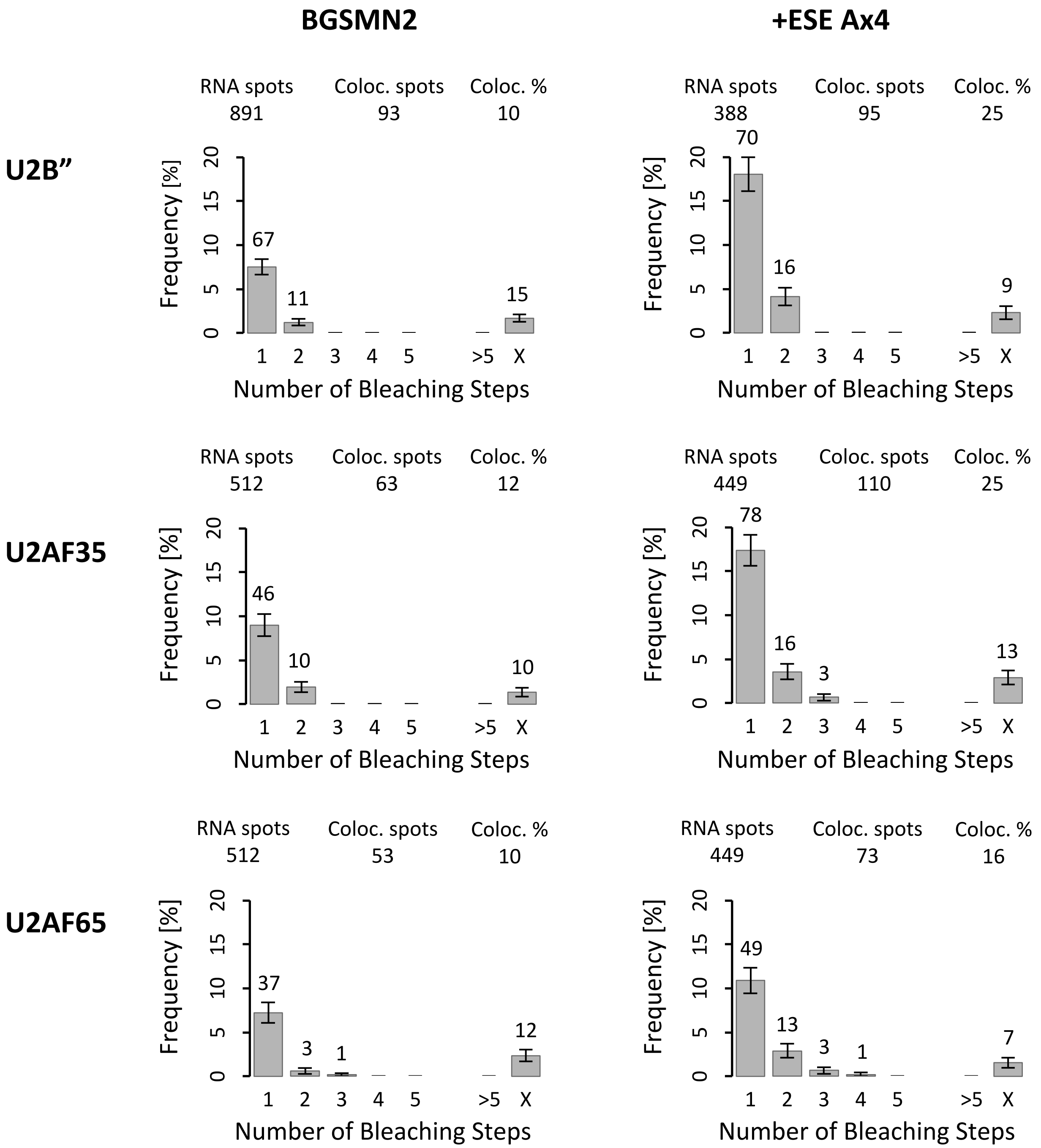

Supplementary Figure S5

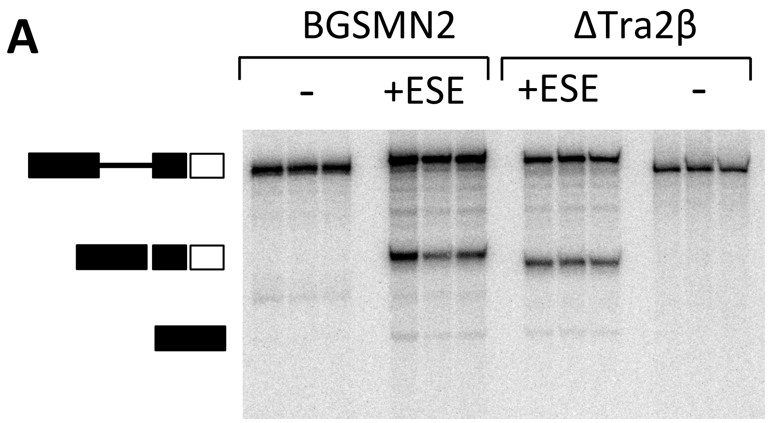

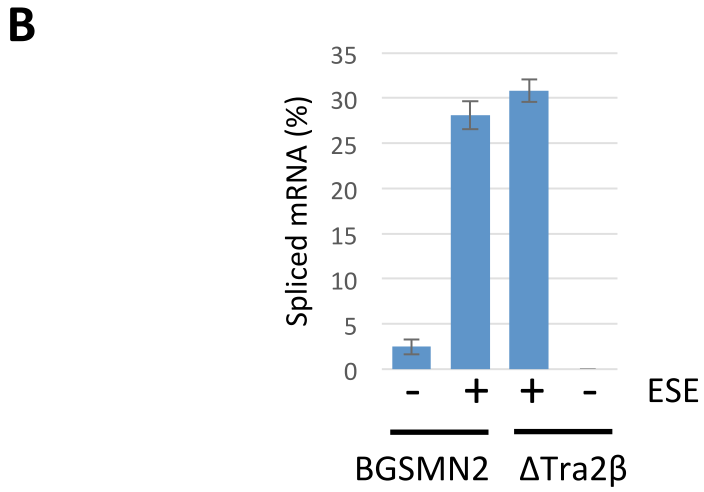

LEGENDS FOR SUPPLEMENTARY FIGURES

Supplementary Figure S1. Frequency histograms showing the relative numbers of complexes in which mEGFP-SRSF1 fluorescence would bleach in one, two, three or four steps if all complexes were occupied at either one site or two sites. This takes account of the observed level of dimerization and the relative levels of mEGFP-labelled and unlabelled SRSF1 in the nuclear extract.

Supplementary Figure S2. Effects of tandemly-repeated ESE-A sequences on splicing and splicing complex formation. (A) Splicing activities *in vitro* of BGSMN2 pre-mRNAs with one to four 3′-terminal copies of ESE-A. Reactions contained the same batch of nuclear extract and were incubated for two hours. The pre-mRNA, mRNA and 5′ exon intermediate are shown. Note that the additional copies of ESE-A affect the lengths of the pre-mRNA and mRNA but not the 5′ exon. (B) analysis of splicing complexes by native gel electrophoresis. After incubation for the times shown (mins) above the lanes, reactions were treated with heparin and analysed by electrophoresis on an agarose gel.

Supplementary Figure S3. The number of protein molecules contacting the ESE increases with the number of repeats of ESE-A. Gel-purified transcripts comprising only a cap nucleotide and one to four copies of ESE-A were incubated in nuclear extract in splicing conditions, irradiated and digested with ribonucleases. Reactions were run on SDS-PAGE and transferred to a nitrocellulose membrane. The membrane was incubated with anti-SRSF1 and fluorescent anti-mouse antibody. (A) Phosphor image of the membrane. (B) Image of fluorescence from SRSF1 (green). (C) Superimposition of the images in (A) and (B) using protein markers detected by colour and located by radioactive registration marks in the phosphor image and fluorescence from fluorescent anti-rabbit antibody in (B). The slightly lower mobility of the cross-linked protein is attributed to the RNA tag left after RNase digestion. Mass spectrometry (Reichenbach et al., in preparation) suggests that the band just above 37 kDa is hnRNP A/B and the one above 50 kDa is hnRNP F/H. (D) Quantification of the radioactivity in the band aligning with SRSF1 (arbitrary phosphor imager units).

Supplementary Figure S4. Frequency histograms showing the effects of ESE sequences on the binding of U2 snRNP, U2AF35 and U2AF65. A nuclear extract expressing mEGFP-U2B'' was used for the measurements of U2 snRNP association and an extract expressing mCherry-U2AF35 and mEGFP-U2AF65 was used for measuring the association of U2AF, as described{Chen, 2017 #3530}.

Supplementary Figure S5. The Tra2β enhancer region is superfluous in the presence of an additional SRSF1-dependent enhancer. (A) Splicing reactions were done for two hours in the presence or absence of ESE-AX2 on pre-mRNA containing (BGSMN2) or lacking (∆Tra2β) the purine-rich binding site for Tra2β. (B) Mean proportions of spliced mRNA, with error bars showing the standard error of the mean.
